# The complete chloroplast genome and phylogenetic analysis of *Guilandina minax* (Hance) G. P. Lewis (Fabaceae)

**DOI:** 10.1080/23802359.2026.2629683

**Published:** 2026-02-23

**Authors:** Qian Zhang, Bowen Liu, Xianglan Liang, Kaiming Gao, JunJie Wang

**Affiliations:** a Xiangnan University-Chenzhou Industrial Technology Research Institute, Xiangnan University, Chenzhou, PR China; b Teaching and Research Section of Surgery, Xiangnan University Affiliated Hospital, Chenzhou, PR China; c School of Basic Medical Sciences, Xiangnan University, Chenzhou, PR China; d School of Chemistry and Chemical Engineering, Guangdong Pharmaceutical University, Guangzhou, PR China; e Institute of Medicinal Plant Development, Chinese Academy of Medical Sciences & Peking Union Medical College, Beijing, PR China

**Keywords:** Chloroplast, *Guilandina minax*, Fabaceae, Phylogenetic analysis

## Abstract

*Guilandina minax* (Hance) G. P. Lewis, commonly known as ‘Whiteflower Cacalia’, is a traditional medicinal plant extensively utilized by the Zhuang, Yao, and Dai ethnic minority groups. In this study, we sequenced and characterized the complete chloroplast genome of *G. minax*, and conducted a phylogenetic analysis to elucidate its evolutionary relationships within the Fabaceae. The chloroplast genome of *G. minax* was found to be 156,769 bp in length, with an overall GC content of 37.20%. It exhibited the typical quadripartite structure comprising a large single-copy (LSC) region of 85,264 bp, a small single-copy (SSC) region of 15,543 bp, and a pair of inverted repeat (IR) regions, each measuring 27,981 bp. A total of 127 genes were annotated from the chloroplast genome of *G. minax*, including 82 protein-coding genes, 37 transfer RNA (tRNA) genes, and 8 ribosomal RNA (rRNA) genes. A maximum-likelihood (ML) phylogenetic analysis based on the complete chloroplast genomes of *G. minax* along with 28 species from the Fabaceae family and 2 outgroup taxa indicated that *G. minax* is most closely related to *Guilandina bonduc*. This study provides valuable genomic resources for *G. minax* and contributes to a more resolved understanding of phylogenetic relationships within the Fabaceae.

## Introduction

*Guilandina minax* (Hance) G. P. Lewis 2020 is a kind of vine medicinal plant of the genus *Guilandina* (Lewis 2020) in the Fabaceae family and is widely distributed throughout tropical and subtropical regions. The seeds of this plant are named ‘ku-shi-lian’ in China and widely used as a traditional medicine for the treatment of rheumatism, dysentery, and the common cold (Jiangsu New Medical College 1986). The chemical research of the seeds of *G. minax* focused on diterpenoids, mainly cassane-type diterpenes (Zheng et al. 2013). Its pharmacological effects cover multiple aspects such as anti-inflammation (Dong et al. 2015), anti-tumor (Xu et al. 2022), anti-bacterial (Linn et al. 2005), anti-virus (Wu et al. 2014), anti-malaria (Ma et al. 2014), and anti-oxidation (Zhang et al. 2015). Therefore, *G. minax* currently shows a promising development prospect.

This species was previously classified under the genus *Caesalpinia* as *Caesalpinia minax* (Dong et al. 2015), but was later reclassified to *Guilandina* based on a recent taxonomic revision (Lewis 2020). Chloroplast genomes are maternally inherited, highly conserved (Korpelainen 2004), and exist in multiple copies per cell, making them powerful tools for species identification, molecular taxonomy, and evolutionary studies (Yamane et al. 2006; Reginato et al. 2016). However, until now, the chloroplast genome of *G. minax* has not been reported, and its phylogenetic position within the Fabaceae family has remained unclear. In this study, we assembled and annotated the complete chloroplast genome of *G. minax* and reconstructed a phylogenetic tree including representative species of Fabaceae. These findings provide a foundational genomic resource for future research on species delimitation, phylogenetic relationships, and the exploration of novel medicinal plant resources.

## Materials and methods

**Figure 1.**
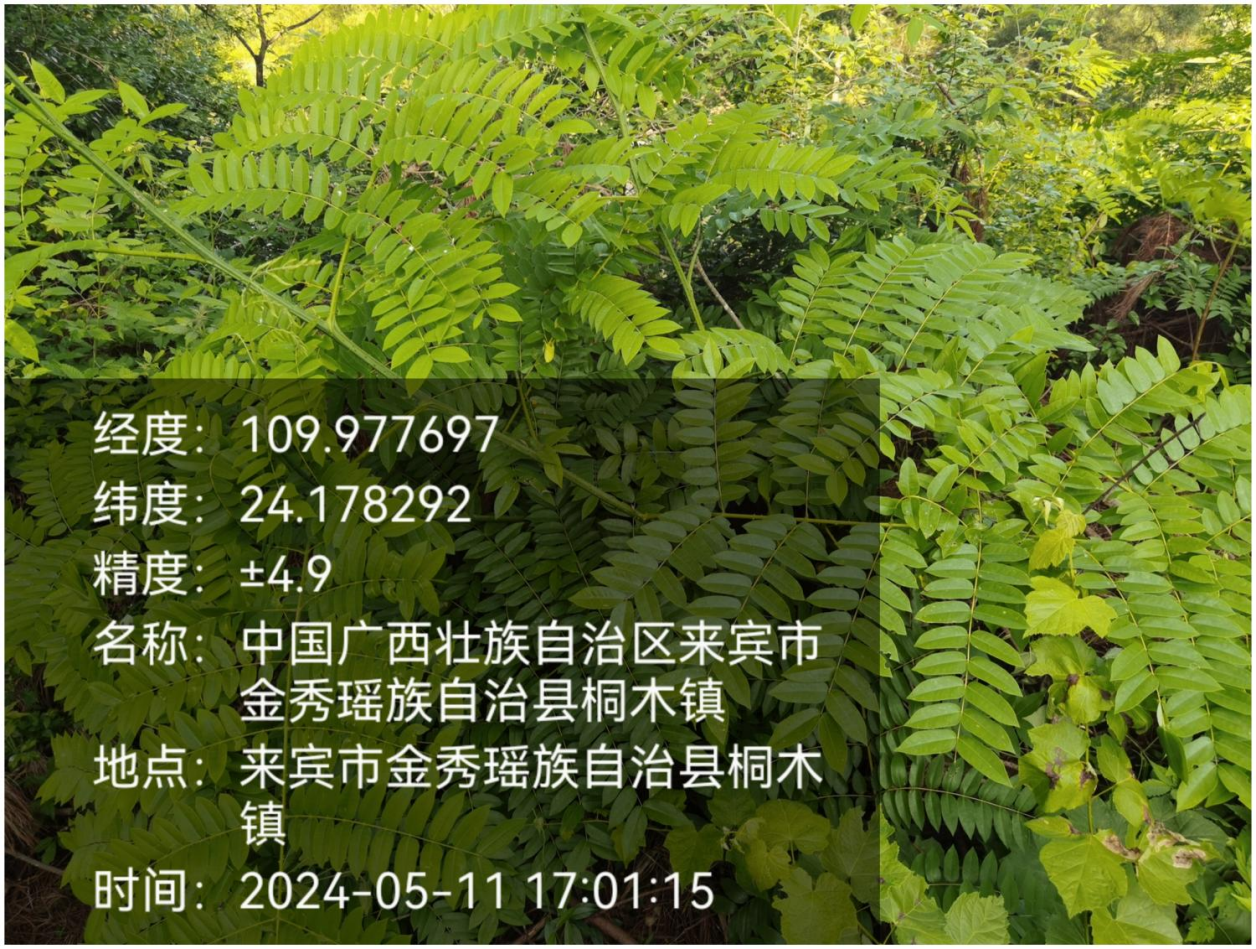
*Guilandina minax*. The photo was taken by Jiahua Chen (825981332@qq.com) in Jinxiu Yao Autonomous County, Guangxi Province, China. The plants are prickly shrubs, pubescent throughout. The stems are covered with needlelike prickles. The leaves are bipinnately compound, and the leaflets are elliptic to oblong in shape. The leaflet apex ranges from obtuse to acute, the base is rounded and slightly oblique. Both surfaces of the leaflets have pubescence along the midvein.

Fresh leaves of *Guilandina minax* were collected from Jinxiu Yao Autonomous County, Guangxi Province, China (24.178°N, 109.978°E) (Figure 1). The plant material was collected by Xinmin Pan (email: 40971972@qq.com) with authorized permission. The voucher specimen (JXHC106) is preserved in the herbarium of the Institute of Medicinal Plant Development.

Total genomic DNA was extracted from leaf tissues using a Plant Genomic DNA Kit (Tiangen, Beijing, China) following the manufacturer’s instructions. The quality, integrity, and concentration of the extracted DNA were assessed by agarose gel electrophoresis and spectrophotometric analysis. The high-quality genomic DNA was fragmented into approximately 300 bp segments for the construction of paired-end (PE) libraries. Sequencing was carried out in 150 bp PE mode using the Illumina HiSeq 2500 platform (Illumina, CA, USA). Raw sequencing reads were subjected to quality filtering with fastp v0.23.2 (Chen 2023), yielding 14.85 Gb of high-quality clean data. The chloroplast genome of *G. minax* was assembled using GetOrganelle v1.7.7.1 (Jin et al. 2020) with the parameters: -t 15 -k 21,65,85,127-R 40-F embplant_pt –reduce-reads-for-coverage inf –max-reads inf. This resulted in the successful reconstruction of a complete circular chloroplast genome. To assess genome coverage, sequencing reads were aligned to the assembled genome using BWA v0.7.17 (Li 2013), and coverage statistics were computed with Samtools v1.13 (Danecek et al. 2021). Chloroplast gene annotation was performed using CPGAVAS2 (Shi et al. 2019), with results summarized and manually reviewed in Apollo v1.11.8 (Lewis et al. 2002); tRNA genes were validated using tRNAscan-SE (Chan et al. 2021). CPGView (Liu et al. 2023) was then employed to generate visual maps of the chloroplast genome and to illustrate the structures of cis- and trans-splicing genes.

To explore the phylogenetic position of *G. minax*, chloroplast genome sequences of 28 related Fabaceae species were obtained from NCBI GenBank, with *Polygala tenuifolia* (MT221251.1) (Lee et al. 2020) and *Monnina hirta* (PP883953.1) serving as outgroups. A total of 31 complete chloroplast genomes, including that of *G. minax*, were aligned using MAFFT v7.505 (Katoh et al. 2002) with default settings. Phylogenetic analysis was conducted using the maximum-likelihood (ML) method implemented in IQ-TREE v2.1.4 (Lanfear et al. 2020), applying the TVM+F + R5 substitution model and 1,000 ultrafast bootstrap replicates. The resulting tree was visualized with the Interactive Tree of Life (iTOL) online tool (https://itol.embl.de/) (Letunic and Bork 2024).

## Results

**Figure 2.**
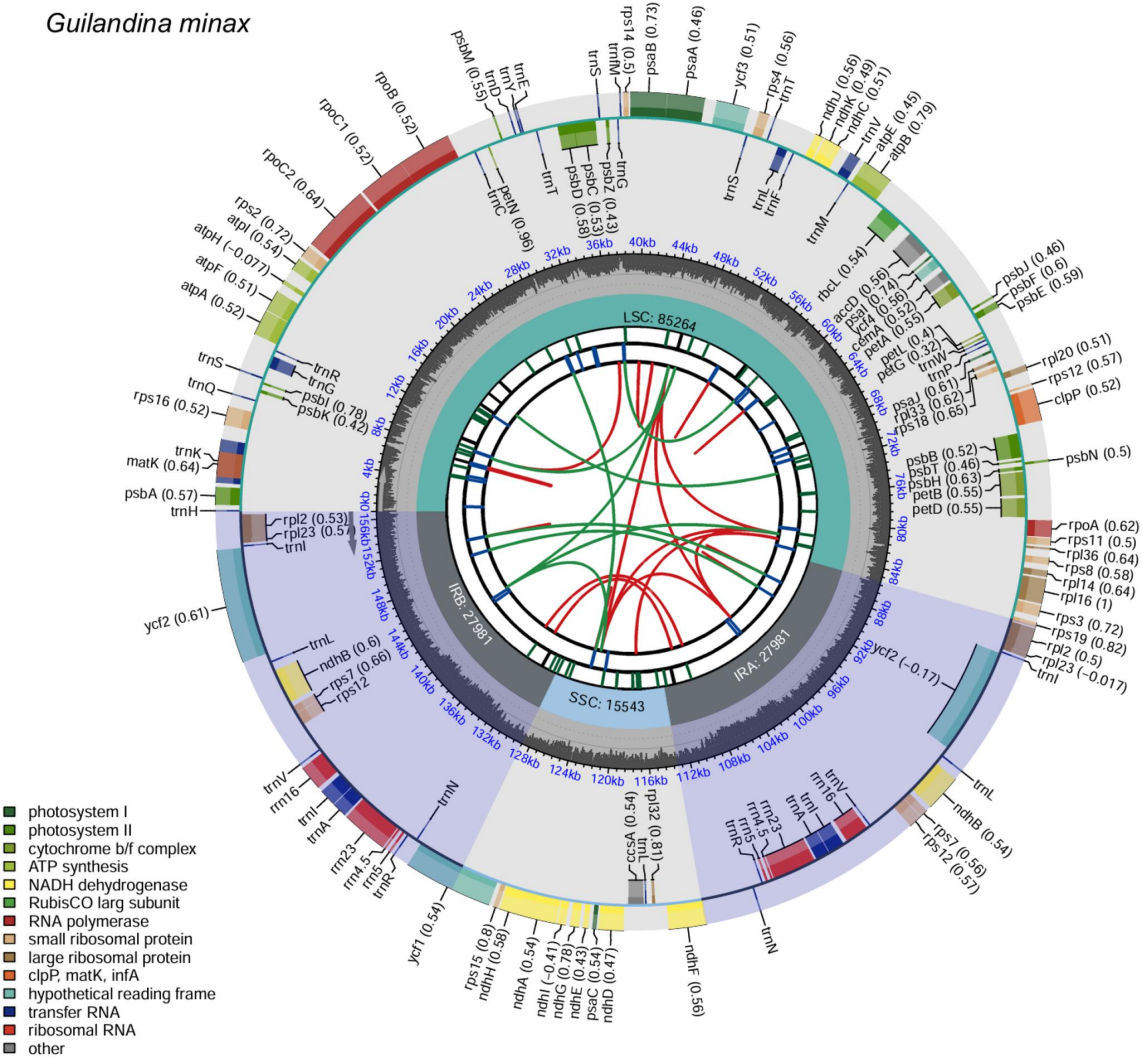
Circular representation of the *G. minax* chloroplast genome generated using CPGview (Liu et al. 2023), comprising six annotated tracks. The innermost track illustrates dispersed repeats, with direct (D) and palindromic (P) repeats visualized as red and green arcs, respectively. The second track marks long tandem repeats with short blue bars, whereas the third track identifies simple sequence repeats (SSRs) or microsatellites using colored bars of variable lengths. The fourth track delineates the major structural regions of the genome, including the large single-copy (LSC), small single-copy (SSC), and a pair of inverted repeats (IRa and IRb). The fifth track represents the GC content across the genome, while the outermost track displays annotated genes, color-coded based on their functional categories. Codon usage bias, where applicable, is indicated in parentheses following gene names. Genes oriented in the clockwise direction are located on the inner side of the circle, while those transcribed in the counterclockwise direction are positioned on the outer side. A legend indicating the functional classification of genes is provided in the lower left corner of the map.

The complete chloroplast genome of *G. minax* is 156,769 bp in length and exhibits a typical quadripartite structure (Figure 2). The assembled genome achieved an average read mapping depth of 10,085.87× (Figure S1), indicating high sequencing coverage and assembly quality. It comprises a large single-copy (LSC) region of 85,264 bp, a small single-copy (SSC) region of 15,543 bp, and a pair of inverted repeat (IR) regions, each measuring 27,981 bp. The overall GC content of the chloroplast genome is 37.20%. Across the chloroplast genome, the IR regions exhibit the highest GC content at 41.70%, followed by the LSC region at 34.24%, while the SSC region has the lowest GC content at 30.76%. A total of 127 genes were annotated in the genome, including 82 protein-coding genes (CDS), 37 transfer RNA (tRNA) genes, and 8 ribosomal RNA (rRNA) genes. Among these, nine genes (*rps*16, *atp*F, *rpo*C1, *pet*B, *pet*D, *rpl*16, *rpl*2, *ndh*B, and *ndh*A) each contain a single intron (Figure S2). Two genes (*clp*P and *ycf*3) harbor two introns, and *rps*12 is trans-spliced (Figure S3).

**Figure 3.**
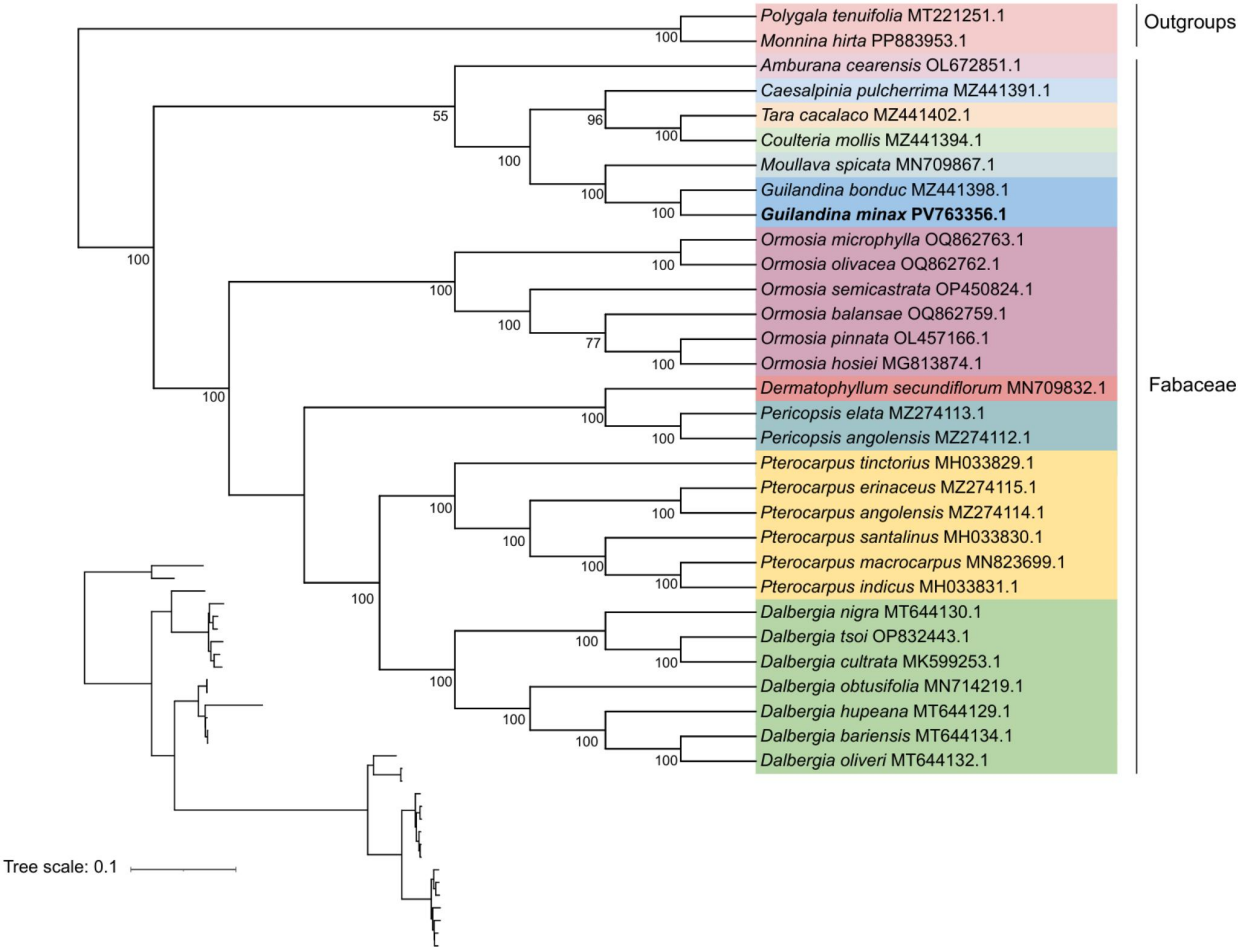
Maximum-likelihood tree based on the chloroplast genomes of *G. minax* and 30 other species. The number next to the nodes indicates the bootstrap values. Species belonging to the same genus within the Fabaceae family are highlighted with the same background color. The position of *G. minax* is marked in bold. The following sequences were used: *G. bonduc* (MZ441398.1), *Amburana cearensis* (OL672851.1) (Choi et al. 2022), *C. pulcherrima* (MZ441391.1) (Aecyo et al. 2021), *Coulteria mollis* (MZ441394.1) (Aecyo et al. 2021), *Moullava spicata* (MN709867.1) (Aecyo et al. 2021), *Tara cacalaco* (MZ441402.1) (Aecyo et al. 2021), *Dermatophyllum secundiflorum* (MN709832.1) (Zhang et al. 2020), *Ormosia semicastrata* (OP450824.1), *Ormosia balansae* (OQ862759.1) (Tang et al. 2023), *Ormosia hosiei* (MG813874.1) (Zhang et al. 2019), *Ormosia pinnata* (OL457166.1), *Ormosia microphylla* (OQ862763.1) (Tang et al. 2023), *Ormosia olivacea* (OQ862762.1) (Tang et al. 2023), *Pterocarpus santalinus* (MH033830.1) (Jiao et al. 2019), *Pterocarpus tinctorius* (MH033829.1) (Jiao et al. 2019), *Pterocarpus indicus* (MH033831.1) (Jiao et al. 2019), *Pterocarpus macrocarpus* (MN823699.1) (Zhang et al. 2020), *Pericopsis elata* (MZ274113.1) (Mascarello et al. 2021), *Pericopsis angolensis* (MZ274112.1) (Mascarello et al. 2021), *Pterocarpus angolensis* (MZ274114.1), *Pterocarpus erinaceus* (MZ274115.1) (Mascarello et al. 2021), *Dalbergia hupeana* (MT644129.1) (Hong et al. 2022), *Dalbergia oliveri* (MT644132.1) (Hong et al. 2022), *Dalbergia bariensis* (MT644134.1) (Hong et al. 2022), *Dalbergia obtusifolia* (MN714219.1) (Li et al. 2022), *Dalbergia cultrate* (MK599253.1) (Liu et al. 2019), *Dalbergia nigra* (MT644130.1) (Hong et al. 2022), *Dalbergia tsoi* (OP832443.1). *P. tenuifolia* (MT221251.1) (Lee et al. 2020) and *M. hirta* (PP883953.1) served as outgroups.

The reconstructed phylogenetic tree, based on 31 complete chloroplast genome sequences, clearly resolved the phylogenetic placement of *G. minax* within the family Fabaceae (Figure 3). Most of the branches in the tree exhibited bootstrap support values of 100%, indicating a highly robust phylogenetic framework. The analysis revealed that *G. minax* is most closely related to *Guilandina bonduc*. At the generic level, *Guilandina* was found to be most closely related to *Moullava*, with *G. minax* and *Moullava bonduc* forming a well-supported sister group.

## Discussion and conclusion

In earlier studies, the species now known as *G. minax* was referred to as *Caesalpinia minax* (Jiang et al. 2001; Ye et al. 2022). *Guilandin*a is a genus characterized by taxonomic complexity and nomenclatural challenges (Gagnon et al. 2016). In a recent taxonomic revision, Lewis (2020) transferred seven species, including *C. minax*, to the genus *Guilandina*. In this study, we conducted a phylogenetic analysis including two species from the genus *Guilandina* (*G. minax* and *G. bonduc*), one from *Caesalpinia* (*Caesalpinia pulcherrima*), and 26 additional species from other genera within the Fabaceae. The results revealed clear phylogenetic distinctions between species of *Guilandina* and *Caesalpinia*, consistent with previously proposed relationships within the *Caesalpinia* group (Aecyo et al. 2021).

To date, only the chloroplast genome of *G. bonduc* (MZ441398.1) has been sequenced and published within this genus. Here, we report the complete chloroplast genome of another species, *G. minax*. The chloroplast genome of *G. minax* shows a similar overall structure to that of *G. bonduc*, with a total length of approximately 156 kb and a GC content of around 37%, consistent with that of *G. bonduc*, further supporting the conserved nature of chloroplast genomes across the genus (Cai et al. 2025; Li et al. 2025).

This newly characterized chloroplast genome of *G. minax* provides a valuable genomic resource for future studies. The genomic features presented here offer meaningful insights that can support taxonomic, evolutionary, and phylogenetic research within *Guilandina* and the broader Fabaceae family.

## Supplementary Material

Supplemental Material

## Data Availability

The genome sequence data that support the findings of this study are publicly available from the NCBI GenBank database under accession number PV763356. The associated BioProject, BioSample, and SRA accession numbers are PRJNA1170859, SAMN44110547, and SRR30940501, respectively.
