# Supplementary material for: The complete chloroplast genome and phylogenetic analysis of Guilandina minax (Hance) G. P. Lewis (Fabaceae)
Source: Mitochondrial DNA B Resour. 2026 Feb 23;11(3):434–9. doi: 10.1080/23802359.2026.2629683 (PMC12931340; doi:10.1080/23802359.2026.2629683)

Figure S1. Coverage profile and sequencing depth across the chloroplast genome assembly of *G. minax*. The X-axis indicates genomic coordinates, while the Y-axis represents the sequencing depth at each nucleotide position.


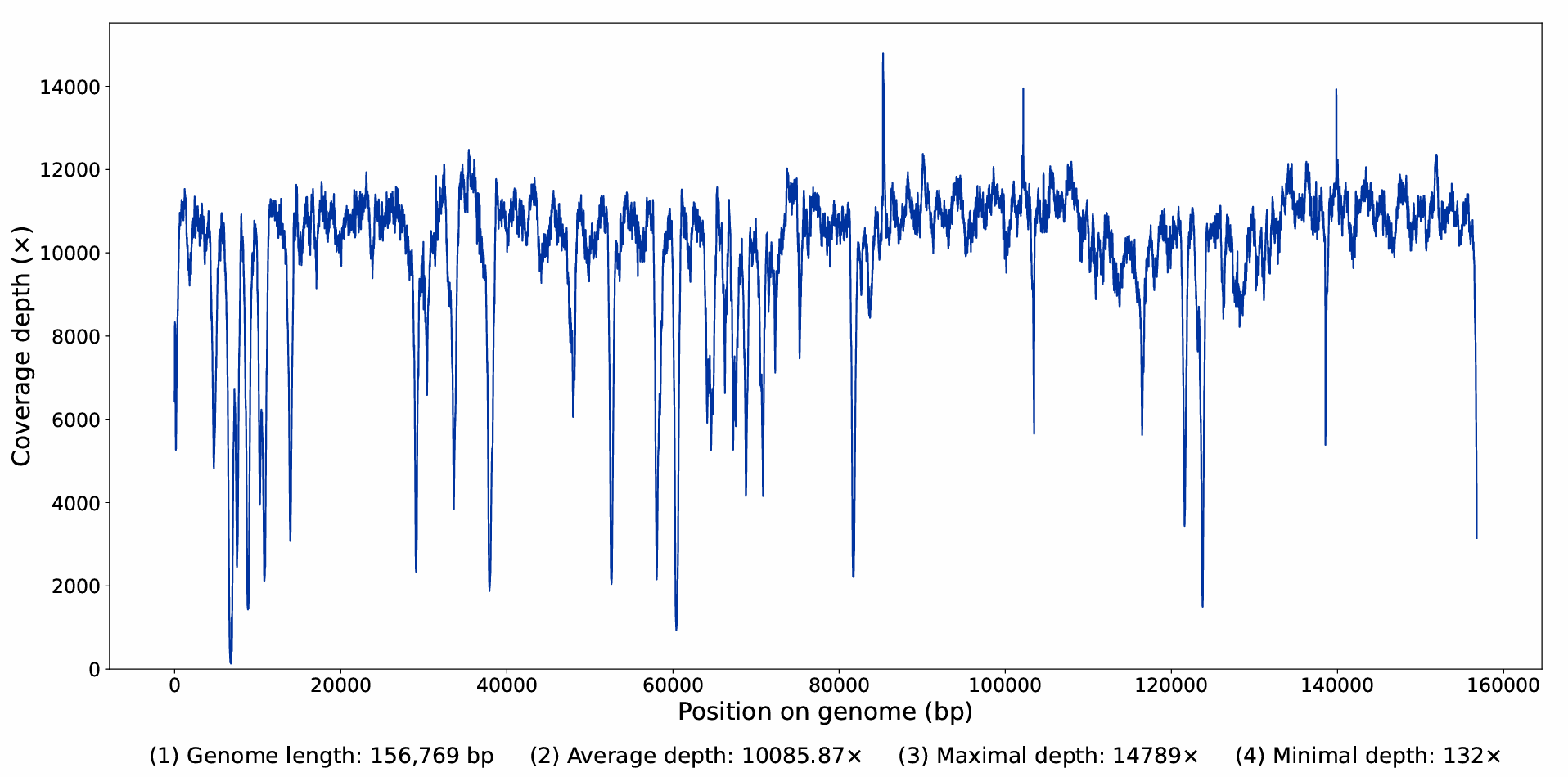


Figure S2. Schematic representation of the cis-splicing genes in the chloroplast genome of *G. minax*. Genes are vertically arranged in genomic order from top to bottom. Gene names are listed on the left, with corresponding gene structures on the right. Exons are depicted as black boxes, while introns are shown as white boxes. Arrows indicate the direction of transcription. Note that the lengths of exons and introns are not drawn to scale.


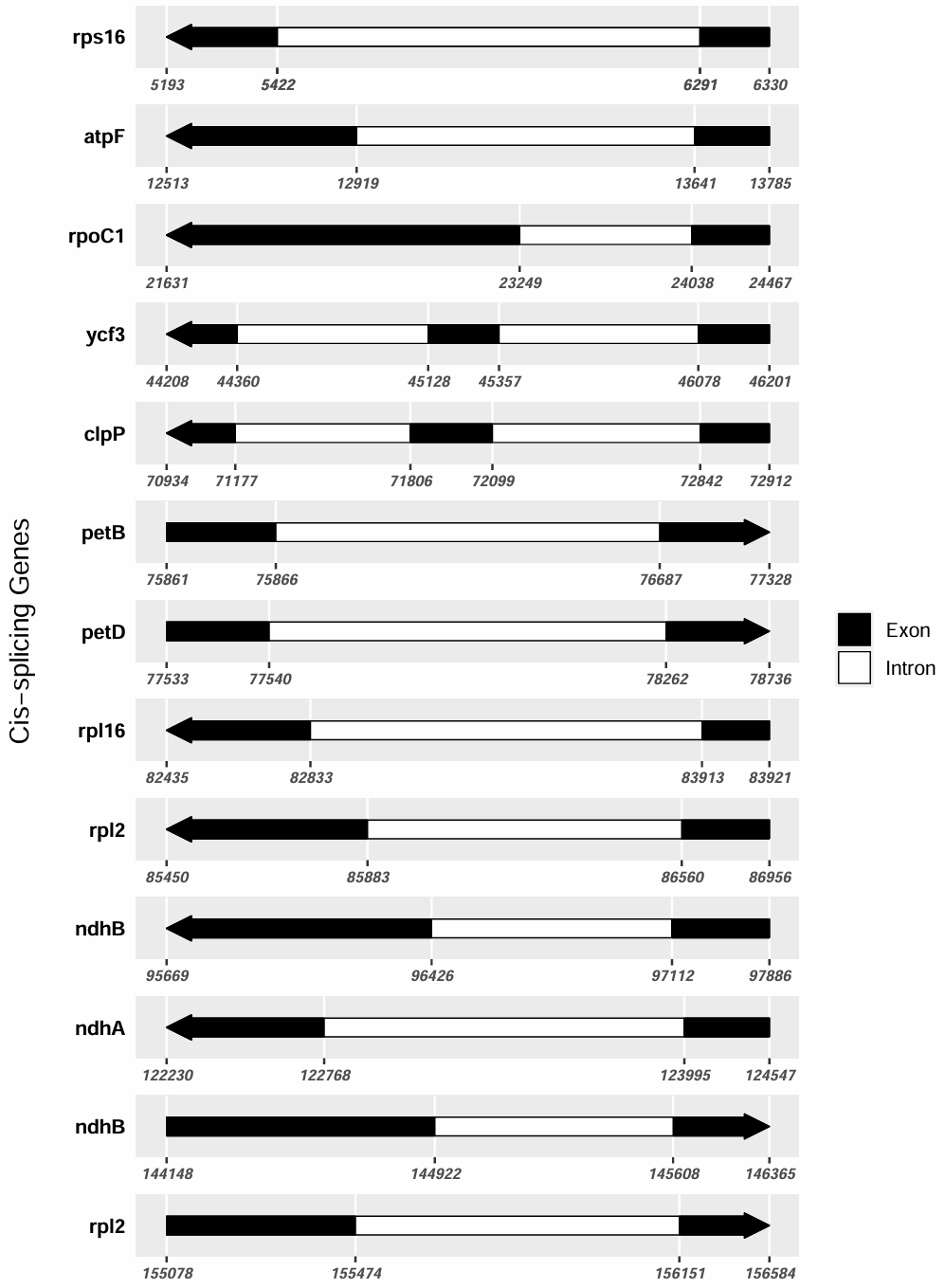


Figure S3. Schematic representation of the trans-spliced gene *rps*12 in the chloroplast genome of *G. minax*. This gene comprises three distinct exons, with two of them duplicated as a result of their presence within the inverted repeat (IR) regions.


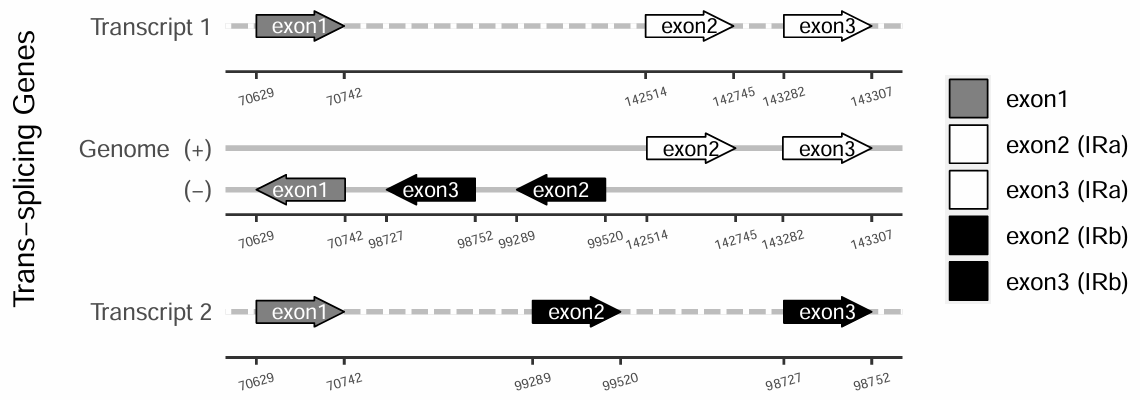

Supplement: Supplemental Material [file TMDN_A_2629683_SM1863.docx]
